# Active Transport and Ocular Distribution of Intravitreally Injected Liposomes

**DOI:** 10.1167/tvst.12.8.20

**Published:** 2023-08-24

**Authors:** Anne Zebitz Eriksen, Fredrik Melander, Grace De Malona Eriksen, Paul Joseph Kempen, Andreas Kjaer, Thomas Lars Andresen, Andrew James Urquhart

**Affiliations:** 1 Department of Health Technology, Technical University of Denmark, Lyngby, Denmark; 2 National Center for Nano Fabrication and Characterization, Technical University of Denmark, Lyngby, Denmark; 3 Department of Clinical Physiology, Nuclear Medicine and PET & Cluster for Molecular Imaging, Copenhagen University Hospital – Rigshospitalet, Copenhagen, Denmark; 4 Department of Biomedical Sciences, University of Copenhagen, Copenhagen, Denmark

**Keywords:** intravitreal drug delivery, retina, liposomes, active transport

## Abstract

**Purpose:**

Drug delivery to the retina remains a challenge due to ocular barriers and fast clearing mechanisms. Nanocarrier drug delivery systems (NDDSs) hold the promise of prolonging intraocular retention times and increasing drug concentrations in the retina.

**Methods:**

Anionic and cationic PEGylated liposomes, loaded with oxaliplatin (OxPt) to be used as trace element, were prepared from dry lipid powders. The differently charged liposomes were intravitreally injected in C57BL/6JrJ mice; eyes were harvested 2 hours and 24 hours post-injection. To investigate active transport mechanisms in the eye, a subset of mice were pre-injected with chloroquine before injection with cationic liposomes. Eyes were dissected and the distribution of OxPt in different tissues were quantified by inductively coupled plasma mass spectrometry (ICP-MS).

**Results:**

Both liposome formulations enhanced the retention time of OxPt in the vitreous over free OxPt. Surprisingly, when formulated in cationic liposomes, OxPt translocated through the retina and accumulated in the RPE-sclera. Pre-injection with chloroquine inhibited the transport of liposomal OxPt from the vitreous to the RPE-sclera.

**Conclusions:**

We show that liposomes can enhance the retention time of small molecular drugs in the vitreous and that active transport mechanisms are involved in the trans retinal transport of NDDS after intravitreal injections.

**Translational Relevance:**

These results highlight the need for understanding the dynamics of ocular transport mechanisms in living eyes when designing NDDS with the back of the eye as the target. Active transport of nanocarriers through the retina will limit the drug concentration in the neuronal retina but might be exploited for targeting the RPE.

## Introduction

Diseases of the retina, such as glaucoma, age-related macular degeneration (AMD), and diabetic retinopathy (DR), are leading causes of acquired blindness. These diseases are on the rise due to aging populations and lifestyle.^1^^,^^2^ Diseases of the retina present a considerable therapeutic challenge. Ocular barriers inhibit drugs from reaching the retina if using conventional administration routes (e.g. systemic administration or eyedrops)^3^ because active transport mechanisms and tear film turnover rapidly clear drugs from the eye. Intravitreal injection, where drugs are injected directly into the vitreous, is the clinically preferred method for bypassing the front of the eye barriers in order to achieve a therapeutic concentration at the retina.^4^ However, this administration route suffers from fast clearance rates and the need for frequent re-administration, leading to problems with adverse effects, for example, retinal tears and retinal detachments.^5^

Intravitreally injected drugs can be cleared either through the anterior route, where fluid is pumped from the posterior chamber to the anterior chamber and drained through the trabecular meshwork and Schlemm's canal,^6^^,^^7^ or through the posterior route through the retina and retinal pigment epithelium (RPE) to the choroid where it is cleared to the blood.^4^ For this reason, nano-sized drug delivery systems (NDDSs) have long been investigated for their potential to prolong the retention time of drugs in the vitreous and enhance delivery to the retina.^8^ Liposomes represent an attractive NDDS for delivery of different relevant drugs to the posterior eye. We have previously shown that liposomes can be loaded with the corticosteroid, prednisolone, as an anti-inflammatory treatment for AMD,^9^ or with biologics to be anti-apoptotic.^10^ Others report liposomes loaded with infliximab as NDDS for against autoimmune uveoretinitis^11^ and the anti-VEGF drug Avastin for wet AMD,^12^^,^^13^ highlighting the versatility and great clinical potential of this delivery platform.

When an NDDS is injected into the vitreous it will meet the vitreous hydrogel that consists of a complex collagen-glycosaminoglycan network, and the size, surface charge, and surface chemistry of the NDDS will be the decisive factors influencing the pharmacodynamics (PDs) of the NDDS.^8^^,^^14^^,^^15^ It is generally accepted that NDDS with diameters <150 nm can diffuse within the vitreous and that positively charged particles, in contrast to negatively charged and neutral particles, get trapped or slowed down by electrostatic interactions.^16^^–^^18^ It is crucial to know the spatiotemporal dynamics of NDDS within the eye (e.g. how fast and how far into the retina the NDDS can penetrate) to predict NDDS biodistribution and pharmacokinetic (PK)/PD. An additional phenomenon that can influence the intraocular distribution of an intravitreally injected NDDS is flow. There is increasing evidence of a posterior directed flow of fluid through the vitreous.^19^ Whether a particle or a molecule is more effected by flow or diffusion is predicted by the dimensionless Péclet number. The Péclet number is defined as Pe = *v*L/D, where *v* is the flow velocity, L a characteristic length, and D is the diffusion constant. When the Péclet number is >1 transportation will be more dependent on flow than diffusion.^19^ The specific Péclet number for a molecule or an NDDS is hard to determine, but in general, increasing particle size will lead to a decrease in the diffusion constant D, and therefore an increase in Pe. This results in a tendency toward a more flow dependent dynamic, that would push the NDDS toward the retina. However, there is still a limited understanding of NDDS ocular distribution, with most in vivo work being qualitative or semiquantitative at best (e.g. following a hydrophobic fluorescent tracer on the carrier system, rather than a hydrophilic cargo molecule etc.).^20^

To overcome the limitation with quantitative data, here, we present an ocular distribution study of two differently charged PEGylated liposomal formulations loaded with oxaliplatin (OxPt) after intravitreal injection in mice. Liposome cargo, OxPt, was quantified in different tissues of the eye using inductively coupled plasma mass spectrometry (ICP-MS) with Pt as the tracer element. We show that cationic liposomes are transported through the retina faster than anionic liposomes or free OxPt. Furthermore, we show that this phenomenon is partially driven by active transport mechanisms involving endosomes and propose that this mechanism of NDDS clearance may be associated with Müller glial cells. Our work highlights the need for more quantitative in vivo studies in the context of the eye, to determine active transport mechanisms that will impact therapeutic efficacy.

## Methods

### Determination of Pt in Liposomes, Cells, and Tissue by ICP-MS

The concentration of lipids and OxPt in liposome samples were determined by ICP-MS quantifying the amount of phosphorous (P) and platinum (Pt) respectively. The P in liposome samples was quantified by diluting liposomes 15,000 times in 2% HCl with 10 ppb gallium (Ga), and comparing them to a standard curve made with known amounts of P.^21^ The lipid concentration was back calculated assuming 35% cholesterol in the final sample. Pt in liposome samples was quantified in a similar manner, diluting liposomes in 2% HCl 0.5 ppb iridium. The level of Pt in tissue and cells was measured according to previously published methods.^22^ In brief, cells or tissue were digested by adding 50  µL 69% NHO_3_ (Sigma) 5  µL 37% HCl (Sigma) and 30  µL 35% H_2_O_2_ and leaving at 60°C overnight. The tissue- or cell-digests were diluted to a total volume of 4 mL in 2% HCl 0.5 ppb iridium and samples were analyzed on an iCAP Q ICP-MS system (Thermo Scientific, DK).

### Preparation of Liposomes

Two liposome formulations loaded with OxPt were prepared, the anionic formulation consisted of 59.8 mol% 1,2-distearoyl-sn-glycerol-3-phosphorcoline (DSPC; Avanti polar lipids) 35 mol% cholesterol (Lipoid), 5 mol% 1,2-distearoyl-sn-glycero-3-phosphoethanolamine-N-[ amino(polyethylene glycol)-2000] (ammonium salt; DSPE-PEG2k; Avanti) and 0.2 mol% 1,2-dipalmitoyl-sn-glycero-3-phosphoethanolamine-N-(lissamine rhodamine B sulfonyl) (ammonium salt; DPPE-RhoB), and the cationic formulation consisted of 49.8 mol% DSPC, 35 mol% cholesterol, 5 mol% DSPE-PEG2k, 0.2 mol% DPPE-RhoB, and 10 mol% 1,2-stearoyl-3-trimethylammonium-propane (chloride salt; DSTAP; Avanti). The liposomes were prepared as previously described.^16^ In brief, stock solutions of the lipids were prepared in 9:1 tert-butanol: milli Q water and the lipid mixtures were prepared and lyophilized to give dry lipid powder. The lipid powders were hydrated with a 15 mg/mL solution of OxPt in 10 mM HEPES 5% glucose at 70°C and extruded through a 100 nm polycarbonate filter before cooling the liposome suspension to room temperature. Non-encapsulated OxPt was removed by a 3-step dialysis over 48 hours against a 1:400 gradient of 10 mM HEPES 5% glucose. The concentration of lipid and OxPt in the final suspensions were measured using ICP-MS, and the size and zeta-potentials were measured on a Malvern Zeta-sizer. Cryo-TEM imaging of liposomes were performed as previously described by Arta et al.^21^

### Liposome Stability

The liposome’s ability to retain the encapsulated OxPt were tested by placing a 100  µL liposome suspension in a 14 KDa dialysis bag and then dialyzed against a 1:40 gradient of 10 mM HEPES 5% glucose at 37 ± 1°C. A sample of 50  µL was taken from the sink volume at different times and replaced with an equal volume of fresh 10 mM HEPES 5% glucose. The concentration of OxPt was then determined by ICP-MS.

### Cellular Uptake of Liposomes and OxPt

The cellular uptake of OxPt loaded liposomes was tested in both mouse retinal endothelial cells (mRECs) and human retinal pigment epithelial cells (ARPE-19). The mREC were cultured on fibronectin coated plates in endothelial cell media (ECM; Innoprot), 5% fetal bovine serum (FBS), 1% ECGS, and 1% penicillin/streptavidin (P/S), whereas ARPE-19 cells were cultured in non-coated plates in Dulbecco's Modified Eagle Media (DMEM) with 10% FBS, 1% P/S. The cells were seeded at 3*10^5^ cell/well in a 6-well plate and incubated for 24 hours at 37°C 5% CO_2_ before adding liposomes diluted with the appropriate media to give a OxPt concentration of 100  µM, in a total of volume of 2 mL/well. The cells were incubated with the liposomes for 4 hours at 37°C 5% CO_2_, before washing the cells 3 times with warm phosphate buffered saline (PBS) to remove non-internalized liposomes. The cells were trypsinized and counted, before pelleting the cells and lysing the cells by addition of 50  µL concentrated nitric acid, 5  µL 32% HCl, and 30  µL 30% H_2_O_2_. The cell lysates were diluted in 2% HCl with 0.5 ppb iridium and Pt content was determined by ICP-MS.

### Cytotoxicity of OxPt Loaded Liposomes

Acute cytotoxicity of the liposomes and the OxPt used as liposome cargo tracer was evaluated in both mREC and ARPE-19 cells using an LDH cytotoxicity assay (CytoTox 96 Non-Radioactive Cytotoxicity assay, Promega), following the instructions of the manufacturer. First, the appropriate cell number was determined from investigating the maximum LDH release from a range of cell densities. A cell density of 15,000 cell/well were found to be optimal. Cells were seeded out in a 96-well plate and incubated for 24 hours. Liposomes or free OxPt was added at a concentration of 100µM OxPt and the cells were then incubated for 24 hours at 37°C 5% CO_2_ before collecting the media in a fresh 96-well plate and adding the CytoTox 96 reagent. The media samples were incubated with the reagent for 30 minutes at room temperature before adding the stop solution and recording the absorbance at 490 nm. Cytotoxicity percentage was found by correcting for background absorbance from the media and spontaneous LDH release, before normalizing to a maximum release control.

### Distribution of OxPt and Liposomes After Intravitreal Injections

All experiments involving animals were carried out in accordance with ARVO statements about the use of animal in vision and ophthalmic research and were pre-approved by the Department of Experimental Medicine at the University of Copenhagen (license number P19-162). Six to 10 weeks old female C57BL/6JrJ mice were kept in a 12-hour light 12-hour dark cycle and given ad libitum access to food and water. The mice were anesthetized with 5% sevoflurane using 30% O_2_ as carrier gas, and the pupils were dilated by adding one drop of 10 mg/mL tropicamide (Mydriacyl) to the corneal surface. Intravitreal injections were performed by making a hole with a gauge 31 needle and cannulating the hole with a <1 mm diameter glass pipette. Then, 2  µL liposome suspension or free OxPt solution with a concentration of 1 mM OxPt was injected. After injection, Geltail was applied to the eyes. Mice were euthanized by cervical dislocation 2 hours or 24 hours post injection, and the eyes were collected in PBS on ice and dissected into cornea, lens, vitreous, retina, and RPE-sclera, within 3 hours. The dissections were carried out under a dissection microscope, starting by separating the cornea from the eye by making a circular cut around the limbus. The lens was removed and the vitreous was then extracted using a pipette, before turning the eyecup inside out using two pairs of tweezers. The retina was then carefully peeled from the visually pigmented RPE layer.

### Active Transport of Liposomes After Intravitreal Injection

To test for active transport of liposomes after intravitreal injection, a subset of 4 mice were pre-injected with 2  µL 0.3 mM chloroquine (Sigma-Aldrich) in PBS, 2 hours prior to intravitreal injection with cationic liposomes. Then, 24 hours after liposome injection the mice were euthanized, and the tissue was collected as described above.

### Statistical Analysis

Statistical analyses were carried out in Graph Pad Prism 9. Students *t*-test, 1-way ANOVA, and post hoc analyses were performed at a significance level of 5%. For the biodistribution, tests were carried out between the different formulations (anionic liposomes, cationic liposomes, and free OxPt) within each tissue. In rare cases where significant differences in standard deviations were found, the data were tested using a Welch correction.

## Results

### OxPt Loaded Liposomes

PEGylated anionic and cationic liposomes loaded with OxPt were successfully prepared.

**Figure 1.**
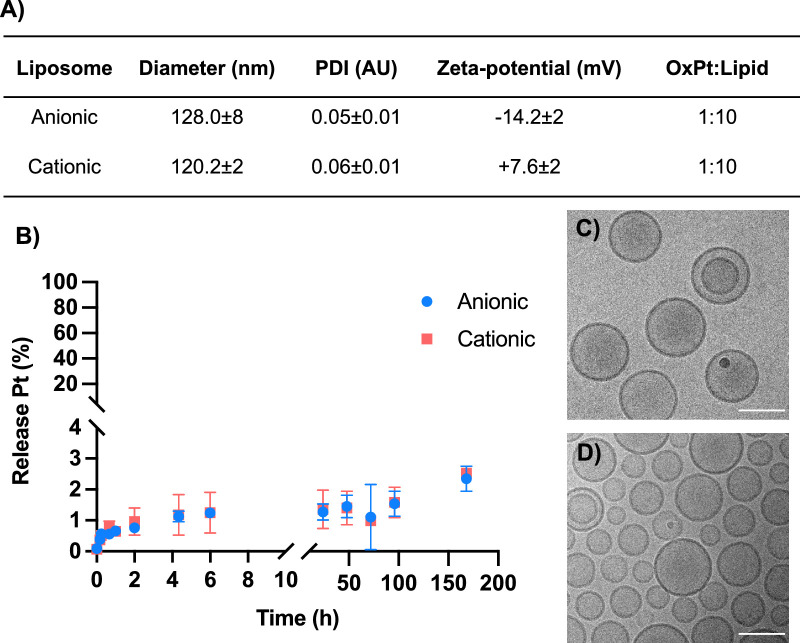
Liposome characterization. (**A**) Liposome characterization table, diameter and PDI measured by dynamic light scattering, mean ± SD (*N* = 2). Zeta potential (mean ± SD) (*N* = 2). OxPt: Lipid after purification by dialysis measured by ICP-MS of phosphor (P) and platinum (Pt). (**B**) OxPt release from liposomes over time at 37°C in PBS. Pt release was measured by ICP-MS of samples taken from sink volume of a dialysis (1:40). (**C**) Cryo-TEM image of anionic liposomes. Scale bar = 100 nm. (**D**) Cryo-TEM image of cationic liposomes. Scale bar = 100 nm.

The two formulations were similar in size and polydispersity index (PDI), 128.0 ± 8 nm and 120.2 ± 2 nm and 0.05 ± 0.01 and 0.06 ± 0.01 for the anionic and cationic formulation, respectively. The anionic formulation had a zeta-potential of −14.2 ± 2 mV, whereas the cationic formulation had a zeta-potential of +7.6 ± 2 mV (Fig. 1A). The formulations had similar encapsulation efficiency resulting in a drug to lipid ratio of 1:10. The liposome stability at 37°C was investigated by following the leakage of OxPt into PBS. The 2 formulations had the same release profile with a small initial burst release over the first hour and then a slow release over the next 200 hours, see Figure 1B. The total release over 200 hours was <3% showing the OxPt remains in the liposome core at physiological temperatures and hence the OxPt is an ideal liposome cargo marker molecule. The liposomes were predominantly unilamellar, as can be seen from the cryo-TEM images in Figures 1C and 1D.

### Liposome Uptake in Cells and In Vitro Cytotoxicity

**Figure 2.**
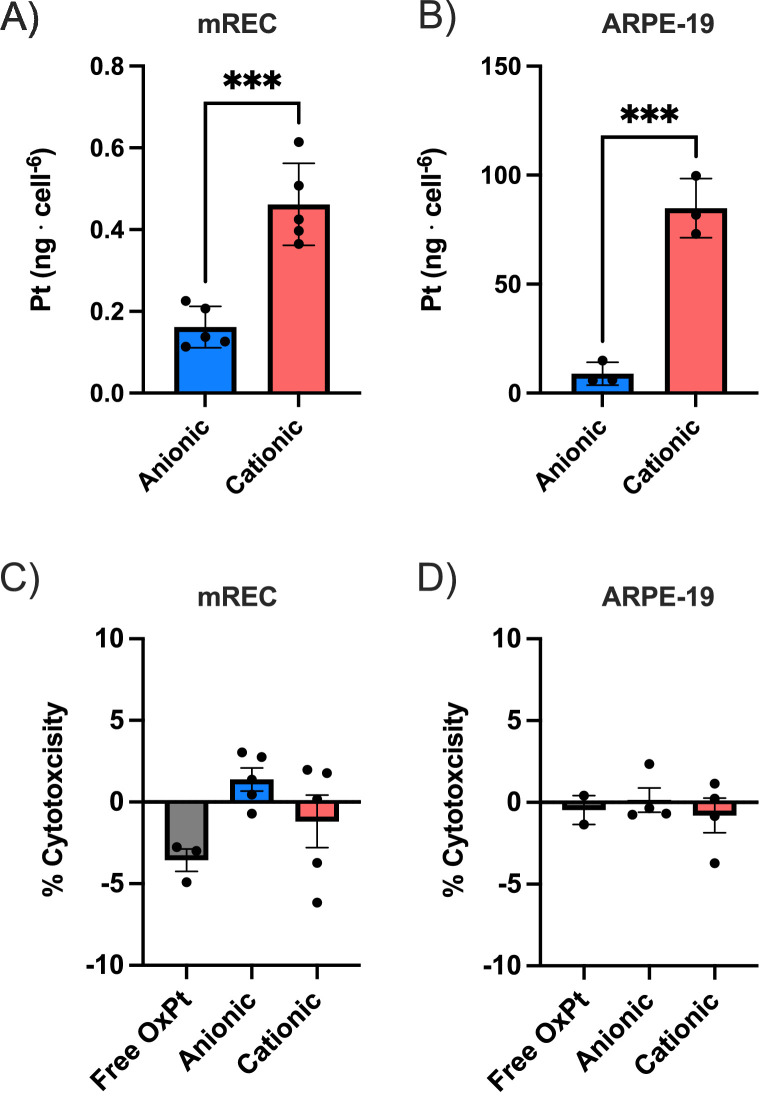
(**A**) Cellular uptake of liposomes in mREC. *Stars* indicate statistical significance, unpaired *t*-test, *P* value = 0.0003. (**B**) Cellular uptake of liposomes in mREC. *Stars* indicate statistical significance, unpaired *t*-test, *P* value = 0.0008. (**C**) Cytotoxicity of OxPt and OxPt loaded liposomes over 24 hours measured by LDH-release in mRECs. (**D**) Cytotoxicity of OxPt and OxPt loaded liposomes over 24 hours measured by LDH-release in ARPE-19 cells.

The in vitro uptake of the anionic and cationic PEGylated liposomes was measured in mREC and ARPE-19 cells. It is generally accepted that positively charged nanocarriers are taken in more efficiently than negatively charged counter parts due to the negative transmembrane potential in most live cells.^23^ The cationic liposomes showed a more than 2-fold higher uptake in vitro in mREC (Fig. 2A) and a 10 times higher uptake in ARPE-19 cells (Fig. 2B) compared to the anionic liposomes. The liposome uptake was almost 250 times higher in the ARPE-19 cells when compared with the mREC, which is likely explained by the phagocytic nature of the APRE-19 cells. The in vitro cytotoxicity of the OxPt loaded liposome was investigated in the two types of retinal cells (mREC and ARPE19) using the LDH assay (Figs. 2C, 2D, respectively). Both liposomes and free OxPt showed negligible cytotoxicity. The short-term low cytotoxicity of the OxPt loaded liposomes is important for the biodistribution experiment as the potential toxic effect of OxPt on ocular tissue can be neglected within the timescale of the in vivo experiments.

### Ocular Distribution of OxPt After Intravitreal Injection

**Figure 3.**
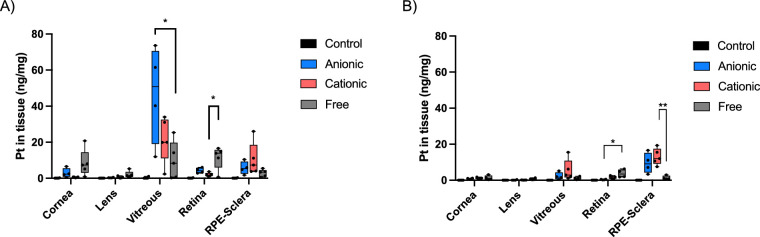
Pt concentration in ng per mg tissue after intravitreal injection of 2  µL 100 mM OxPt formulated as free drug, in anionic liposomes or in cationic liposomes. Control corresponds to contralateral not-injected eyes (*n* = 5). (**A**) At 2 hours post injection. The *star* indicates statistical significance (vitreous: 1-way ANOVA adjusted *P* value = 0.025, retina: Welch ANOVA adjusted *P* value = 0.025). (**B**) At 24 hours post injection. The *star* indicates statistical significance (Retina: Welch ANOVA adjusted *P* value = 0.025, RPE-Sclera: 1-way ANOVA adjusted *P* value = 0.007).

The in vivo biodistribution of free OxPt and OxPt loaded liposomes was investigated by intravitreal injection of equal concentrations of OxPt into C57BL6/JrJ mice. Two hours after injection, free OxPt was distributed equally among the vitreous, retina, and cornea (Fig. 3A), whereas liposomal OxPt was predominantly found in the vitreous. Anionic liposomes had a statistically significant higher concentration in the vitreous compared to free OxPt (see Fig. 3A). This confirms that the smaller molecular weight object (i.e. OxPt) is cleared from the vitreous faster than nanocarriers.^4^ Interestingly, free OxPt had the highest transient concentration in the retina, whereas the higher concentration of OxPt in the cornea and lens suggests that the anterior route of clearing is more pronounced for the free OxPt compared to liposomes. The OxPt concentration in the contralateral, non-treated, control eye was below detection in all cases.

At the 24 hour timepoint, the concentration of the free OxPt was still highest in the retina although not significantly higher than the liposomal OxPt, see Figure 3B. The concentration in the vitreous was highest for the liposomal OxPt and a small tendency of better retention of the cationic liposomes in the vitreous was observed, although not significant. Interestingly, the concentration of OxPt from cationic liposomes were significantly higher in the RPE-sclera compared to the free OxPt even though the concentration of this formulation is lower in the retina and the concentration in the vitreous is higher. This suggests that the liposomes are transported faster through the inner retina layers, than the free drug, until they meet the barrier of RPE layer.

### Active Transport of Liposomes Through the Retina

**Figure 4.**
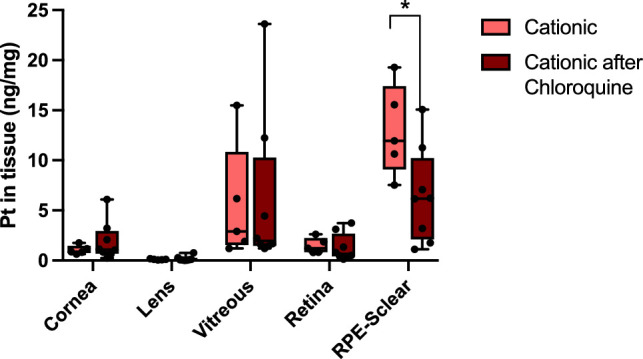
Pt concentration in ng per mg tissue 24 hours after intravitreal injection of 2  µL 100 mM OxPt formulated as cationic liposomes in naïve eyes or eyes preinjected with the cell trafficking inhibitor chloroquine (*n* = 4). The *star* indicates statistical significance, Students *t*-test, *P* value = 0.03.

To test our hypothesis of active transport of liposomes through the retina after intravitreal injection, we pre-injected chloroquine in the vitreous of the eyes of the mice 2 hours prior to injection with cationic liposomes. Chloroquine inhibits clathrin-mediated endocytosis as well as interfering with endosome trafficking.^24^^,^^25^ We chose the cationic formulation because this formulation exhibited a significantly higher in vitro uptake when compared with the anionic formulation, and showed the highest concentration in the RPE-sclera following intravitreal injection. The concentration of cationic liposome cargo OxPt in the RPE-sclera fraction was significantly reduced when clathrin-mediated and endosomal dependent active cellular trafficking mechanisms were inhibited (Fig. 4). Moreover, cargo OxPt concentration was similar in all other tissues independent of chloroquine. This confirms that active transport of liposomes through the cells in the retina is partly responsible for the accumulation of the liposomal cargo in the RPE-sclera.

## Discussion

The size and surface characteristics of NDDS has long been recognized as important parameters, influencing how the NDDS will distribute after intravitreal injection. Equally, the vitreous network has been proposed as a barrier in itself.^18^^,^^26^ The size of the nanocarrier needs to be small enough that it can move through the pores in the vitreous, reported to be between 1 µm and 500 nm^17^ in the bovine vitreous, which is considered a good model of the human vitreous.^27^^–^^29^ Particles larger than 500 nm have been reported to be trapped or show very limited diffusion in the vitreous^17^ and most reports involving nanocarriers for intravitreal injection have diameters <300 nm.^14^ However, reposts suggest that small nanocarriers (<25 nm) are cleared faster than larger nanoparticles, (>200 nm) and most likely through the anterior route.^30^

Another important physicochemical property of nanocarriers is surface chemistry and surface charge. The vitreous is reported to have an overall negative charge^18^ and particles with strong positive surface charges have been reported to become electrostatically trapped in the hydrogel network.^17^^,^^18^^,^^28^^,^^31^^–^^35^ We^16^ and others^20^ have shown that PEGylated liposomes with moderate positive surface charges can move in the vitreous, although more slowly than their negatively charged counter parts, and transfection studies with nanoparticles performed in rats, suggest that a positive charge can be beneficial for retinal uptake of particles and transfection of retinal cells.^36^^,^^37^ This is supported by the recent report by Christensen et al*.* who showed that cationic PEGylated liposomes adsorbed better to the retina in ex vivo porcine eye than anionic PEGylated liposomes.^38^ Here, we tested PEGylated liposomes that were 128.0 ± 8 nm and 120.2 ± 2 nm, hence in a size range that can be expected to diffuse in the vitreous, while decreasing the clearance rate through the anterior segment. We did not observe a significant difference in vitreous and retina distribution of both anionic and cationic liposomes. The lack of an effect of surface charge can likely be attributed to the very limited vitreous in mice, which is approximately 1000 times smaller than in humans.^39^^,^^40^ At the 24-hour timepoint, this biodistribution is altered (albeit as a trend rather than significant) and we observe a higher Pt concentration in the vitreous from the cationic liposomes compared with anionic liposomes and free OxPt. This likely reflects a slower diffusion, or more pronounced trapping, of the cationic liposomes in the negatively charged central vitreous hydrogel.

We tested the liposome stability in vitro in respect to their OxPt release in buffer and found that the liposomes only leak up to approximately 2.5% of their OxPt cargo at 37°C. Liposomal stability, however, might be affected by the proteins and salts present in the vitreous. Our previous study on similar liposomes, using fluorescent correlation spectroscopy (FCS) of both a hydrophobic membrane bound fluorophore and a hydrophilic fluorophore in the liposome core, showed that the liposomes remain intact when diffusing in the porcine vitreous,^16^ and we therefore expect that the liposomes will retain their OxPt cargo when diffusing through the vitreous.

The incorporation of cationic lipids has been reported to increase the cytotoxicity of liposomes.^41^^,^^42^ Here, we compared the cytotoxicity of the two liposome formulations in two retinal cells line (mREC and ARPE-19) and found no significant difference in the cytotoxic response, even though the ARPE-19 cells had an almost 250 times higher uptake of the liposomal Pt, compared to the mREC. No toxic response was observed in the cells within the 24-hour timeframe. The lack of cytotoxicity is likely explained by the mechanism of action of OxPt, involving DNA damage and blockage of DNA repair,^43^ as these effects will mainly be observed in relation to cell division, not likely to happen within 24 hours. The absence of cytotoxicity at the 24-hour time point is important in ensuring that uptake and cellular transport of the liposomes is performed by healthy cells in vivo. Similar cationic liposomes were reported by Christensen et al*.* to be well tolerated by the retina.^38^

We compared the distribution of intravitreally injected free OxPt and liposomal OxPt at 2 hours and 24 hours. In line with previous literature, we observed that the small molecule free OxPt cleared faster than the liposomal OxPt,^44^ showing that even in the small volume vitreous of mice, a positive effect of enhanced retention of nanocarrier encapsulated small molecules can be observed. Interestingly, despite a higher retention in the vitreous, the concentration in the retina remained relatively low after intravitreal injection with liposomes, whereas the concentration in the RPE-sclera fraction increased simultaneously with clearing from the vitreous. The intercellular spacing in the retina is estimated to be <20 nm,^45^ hence smaller than the liposomal diameter. This suggests that active transport mechanisms may be involved in the trans-retinal clearing of liposomes, whereas free OxPt being significantly smaller, could possibly diffuse passively through this route.

When active uptake was inhibited by pre-injection of chloroquine, we observed a significant decrease in the OxPt concentration in the RPE-sclera and a slight increase in other tissues, for example, the vitreous and cornea. The malaria drug chloroquine is a broad inhibitor of clathrin-mediated endocytosis, recognized as the most important cellular uptake mechanism of nanocarriers.^25^^,^^46^ Additionally, chloroquine inhibits endosome trafficking, disrupting intra-cellular transport by increasing lysosomal pH,^24^ and disorganizing the golgi apparatus.^47^ High concentrations of chloroquine have been found to be toxic to photoreceptors after intravitreal injection in cats.^48^ However, no effect was observed on the inner limiting membrane (ILM) or Müller glial cells.^49^ The low concentrations used in this work is not expected to result in any retinal toxicity.^50^^,^^51^

We propose that clathrin mediated uptake and endosomal trafficking is involved in the transport of liposomes from the posterior vitreous. It has been argued that NDDSs are too large to penetrate through the finer protein network of the ILM,^29^ an effect that might be less pronounced in the rodent eye, because of the thinner ILM in mice compared to humans and larger animals.^52^^,^^53^ An important component on the ILM, however, is the end-feet of the Müller glial cells, that span the whole length of the retina from the ILM to the RPE.^20^^,^^54^ Koo et al*.* showed that fluorescently labeled polymeric nanoparticles found, within retinal layers, were located inside the cell bodies of Müller cells.^55^ Müller glial cells are phagocytotic cells,^56^^–^^58^ and are the most abundant glia cells in the retina and act as the main transport cell in the retina (transporting nutrients, ions, water, and waste products to ensure retinal homeostasis).^59^ Müller glial cells can also phagocytose material larger than 500 nm, transporting it to the outer retina.^14^ Interestingly, Müller glial cells in the rabbit were found to be able to penetrate the ILM and endocytose intravitreally injected carbon nanoparticles with a diameter of 20 nm,^60^ and large (1  µm diameter) egg-lecithin coated silicone particles were also found to be phagocytosed by the Müller glial cells.^61^ The chloroquine data presented here indicate that our liposomes are most likely transported through an endocytosis/transcytosis pathway rather than by phagocytosis.

Glial cells are enriched in secretory lysosomes,^62^ and we speculate that it is via this route (liposome endocytosis followed by secretion) that liposomal Pt is transported to the RPE-sclera. If this is the case, then this may be a major clearing mechanism for NDDS in the eyes, but further experiments are needed to conclude this. However, Müller glial cell mediated transport of nanocarriers to the RPE-sclera may provide a route to target diseases associated with RPE breakdown, such as AMD. The observed active transport of the Pt loaded liposomes highlights the need for in vivo evaluations of NDDS intended for intravitreal injection. Whereas it is recognized that the transport dynamics of NDDS are likely slower in the human eye compared with the mouse eye as a result of the size difference, many promising therapies (e.g. cell transplantations^10^ and gene-replacement therapies)^63^^,^^64^ are first tested in mice and a better understanding of the active transport mechanisms of NDDS in the murine retina can aid the development of these novel treatments.

## Conclusions

In this study, we produced both anionic and cationic liposomes loaded with OxPt in order to use Pt as tracer molecule to follow the biodistribution of liposomes after intravitreal injection in mice. Free OxPt distributed equally between the cornea, vitreous, and retina 2 hours post-injection, whereas liposomal OxPt exhibited slower clearing from the vitreous and was predominantly found in the posterior eye (vitreous, retina, and RPE-sclera). The highest OxPt concentration in the retina was observed for free OxPt, whereas cationic liposomes were translocated to the RPE-sclera. We determined that active transport mechanisms were involved in the translocation of liposomes from the vitreous to the RPE-sclera, and that the transport through the retina could be inhibited by the broad functioning uptake inhibitor chloroquine. This active transport plays an important role for the biodistribution of liposome drug delivery systems in the eye and should be considered when designing these systems. Furthermore, it may be possible to exploit these transport mechanisms to target the RPE.
